# Sources of Distress and Coping Strategies Among Emergency Physicians During COVID-19

**DOI:** 10.5811/westjem.2021.9.53406

**Published:** 2021-10-27

**Authors:** Erin Dehon, Kori S. Zachrison, Jennifer Peltzer-Jones, Ramin R. Tabatabai, Elizabeth Clair, Michael A. Puskarich, Amy Ondeyka, Katherine Dixon-Gordon, Lauren A. Walter, Elaine H. Situ-LaCasse, Megan L. Fix

**Affiliations:** * University of Mississippi Medical Center, Department of Emergency Medicine, Jackson, Mississippi; † Massachusetts General Hospital, Department of Emergency Medicine, Boston, Massachusetts; ‡ Henry Ford Health System, Department of Emergency Medicine, Detroit, Michigan; § Keck School of Medicine of USC, Department of Emergency Medicine, Los Angeles, California; ¶ Hennepin Healthcare, Department of Emergency Medicine, Minneapolis, Minnesota; || Inspira Health Network, Department of Emergency Medicine, Vineland, New Jersey; # University of Massachusetts, Psychological and Brain Sciences, Amherst, Massachusetts; ** University of Alabama at Birmingham, Department of Emergency Medicine, Birmingham, Alabama; †† Banner University Medical Center – Tucson, Department of Emergency Medicine, Tucson, Arizona; ‡‡ University of Utah School of Medicine, Department of Emergency Medicine, Salt Lake City, Utah

## Abstract

**Introduction:**

The coronavirus disease 2019 (COVID-19) pandemic has been shown to increase levels of psychological distress among healthcare workers. Little is known, however, about specific positive and negative individual and organizational factors that affect the mental health of emergency physicians (EP) during COVID-19. Our objective was to assess these factors in a broad geographic sample of EPs in the United States.

**Methods:**

We conducted an electronic, prospective, cross-sectional national survey of EPs from October 6–December 29, 2020. Measures assessed negative mental health outcomes (depression, anxiety, post-traumatic stress, and insomnia), positive work-related outcomes, and strategies used to cope with COVID-19. After preliminary analyses and internal reliability testing, we performed four separate three-stage hierarchical multiple regression analyses to examine individual and organizational predictive factors for psychological distress.

**Results:**

Response rate was 50%, with 259 EPs completing the survey from 11 different sites. Overall, 85% of respondents reported negative psychological effects due to COVID-19. Participants reported feeling more stressed (31%), lonelier (26%), more anxious (25%), more irritable (24%) and sadder (17.5%). Prevalence of mental health conditions was 17% for depression, 13% for anxiety, 7.5% for post-traumatic stress disorder (PTSD), and 18% for insomnia. Regular exercise decreased from 69% to 56%, while daily alcohol use increased from 8% to 15%. Coping strategies of behavioral disengagement, self-blame, and venting were significant predictors of psychological distress, while humor and positive reframing were negatively associated with psychological distress.

**Conclusion:**

Emergency physicians have experienced high levels of psychological distress during the COVID-19 pandemic. Those using avoidant coping strategies were most likely to experience depression, anxiety, insomnia, and PTSD, while humor and positive reframing were effective coping strategies.

## INTRODUCTION

Prior to the coronavirus 2019 (COVID-19) pandemic, physicians struggled with heightened levels of burnout, job dissatisfaction, depression, post-traumatic stress symptoms (PTSS), and suicidal ideation.^1^,^2^ Over the past year, emergency physicians (EP) were positioned as frontline caregivers for COVID-19, which further escalated challenges and pressure on the healthcare system and its workers.

Studies have shown that pandemics such as severe acute respiratory syndrome (SARS) 2003 and COVID-19 are associated with increased levels of healthcare worker psychological distress, including burnout, anxiety, depression, insomnia, and post-traumatic stress.^3^–^10^ During the early stages of COVID-19, distress was particularly high in healthcare workers without consistent access to personal protective equipment (PPE)^11^ and those exposed to COVID-19 patients.^12^ A systematic review of 59 internationally diverse studies revealed that psychological distress associated with COVID-19 is a global problem.^13^ Studies of EPs, in particular, show increased levels of psychological distress in response to COVID-19.^10^,^14^,^15^ One survey of over 400 EPs revealed increases in work stress, home anxiety, emotional exhaustion, and burnout.^14^

Given that physicians are experiencing negative effects from the COVID-19 pandemic, it is critical to identify factors influencing physician stress for appropriate interventions to be designed. To date, there is limited data on which interventions have yielded the most success. Of the few published qualitative studies that have investigated potential contributors to physician anxiety, organizational factors such as access to PPE, exposure to COVID-19 at work, uncertainty of organizational support and lack of access to testing, childcare access and up-to-date information and communication were noted as main drivers.^16^

Current EP-specific literature is limited. Most studies were performed outside the US or in limited geographical areas such as New York City. Additionally, many do not include measures of psychological distress with strong validity evidence. Furthermore, there is not, to our knowledge, any current data focusing on possible *positive* psychological reactions to COVID-19 or effective coping strategies. Finally, although some studies have looked at factors contributing to clinician stress, none have performed a comprehensive stepwise approach using an assessment of multiple contributory factors. Our aim in this study was to extend prior research by identifying both individual and organizational factors that place EPs at risk for psychological distress during COVID-19. Additionally, we sought to identify any positive effects related to COVID-19 and examine coping strategies used by EPs.

## METHODS

### Study Design

This was a prospective cross-sectional survey of EPs administered via email between October 6–December 29, 2020. Demographic and work-related data were collected from respondents. We assessed negative mental health outcomes, positive work-related outcomes, and strategies used to cope with COVID-19. All surveys were completed anonymously. This study was approved by the local institutional review board and is reported according to the Strengthening the Reporting of Observational Studies in Epidemiology (STROBE) guidelines (Appendix 1).^17^

**Population Health Research Capsule**

What do we already know about this issue?

*Coronavirus disease 2019 (COVID-19) has led to increased distress among healthcare workers.*

What was the research question?

*What are the factors that place emergency physicians (EP) at risk for psychological distress during COVID-19?*

What was the major finding of the study?

*Coping strategies predicted which EPs experienced distress during COVID-19.*

How does this improve population health?

*Hospitals should support EPs through promoting adaptive coping strategies.*

### Participants and Recruitment

Participants consisted of attending physicians who worked in an emergency department (ED) in the US during the COVID-19 pandemic. To recruit participants, we used a combination of convenience and purposive sampling strategies. A purposive sampling strategy was used to obtain a sample of EPs working in various US regions. We sent directed emails to a convenience sample of known colleagues who work at the identified hospitals asking them to function as survey champions by distributing the survey to all known EPs at their site who had worked in the ED during COVID-19. All participants received a $40 gift card for completing the survey.

### Survey Measures

Below is a brief list of the measures used in this study. For further detail on the measures, please visit Appendix 2.

#### Demographic, Living Arrangements, and Time-of-survey Variables

- Demographics (eg, gender, age, marital status, living arrangements, geographic location)
- Time of survey/day of survey completion was recorded
- Living arrangements

#### COVID-related Variables

##### Pandemic Factors

- Current surge: Was the hospital experiencing a surge at the time of the survey?
- Perceived stigma and interpersonal avoidance – using a measure from SARS 2003 with previously sufficient validity evidence^18^
- Job stress – using a measure from SARS 2003 with previous sufficient validity evidence^18^
- Adequacy of training, protection, and organizational support – using a measure from SARS 2003 with previous sufficient validity evidence^18^
- Current and prior access to PPE

##### Individual Factors

- Fear of COVID-19 infection – using a subscale from a SARS 2003 measure with previous sufficient validity evidence^19^
- Obsession with COVID-19 Scale (OCS)^20^
- Coping with COVID-19 – using the Brief Cope, which assesses both approach and avoidance coping responses^21^

#### Mental Health Outcomes

- Influence of COVID-19 on mental health and daily activities (eg, overall impact on mental health and changes in stress, anxiety, sadness, irritability, loneliness, burnout,^22^ motivation, substance use, social support, and exercise frequency)
- Positive work-related outcomes as a result of COVID-19 – using individual items based on post-traumatic growth and meaning at work measures^23^,^24^ (items were examined individually and not combined to yield a total score)
- Depression – using the Patient Health Questionnaire (PHQ-9)^25^
- Anxiety – using the Generalized Anxiety Disorder-7 (GAD-7)^26^
- Post-traumatic stress symptoms (PTSS) - using the post-traumatic stress disorder (PTSD) checklist for *Diagnostic and Statistical Manual of Mental Disorders*, 5^th^ Edition DSM-5 (PCL-5)^27^
- Insomnia – using the Insomnia Severity Index^28^

### Data Analysis

An a priori power analysis using G*Power^29^ software (University of Dusseldorf, Germany) indicated that the sample size needed to detect a medium effect was 194 based on an alpha of .05, power of .95, and 14 predictors. Preliminary analyses were conducted to test the assumptions for the regression analyses. We calculated response rate using American Association for Public Opinion Research (AAPOR) response rate 2 definition, which allows for the inclusion of both complete and partial surveys.^30^ Non-response bias was evaluated by comparing the early and late participants’ scores on mental health outcomes.^31^ For all measures, we evaluated internal reliability using Cronbach’s alpha. Construct validity was established by examining correlations with other theoretically related, psychological-outcome measures. Basic descriptive statistics and established cutoff scores, and diagnostic algorithms were used to examine the prevalence of PTSD, insomnia, depression, and anxiety among EPs. We used Mann-Whitney U tests to compare psychological outcomes among EPs by demographic and epidemic-related factors. To balance Type I and Type II error across the eight analyses for each of the three outcomes, we applied a Holm-Bonferroni correction.^32^,^33^

We performed four separate three-stage hierarchical multiple regression analyses to examine whether individual and organizational COVID-19 challenges related to COVID (eg, fear of COVID-19, PPE access) were predictive of psychological distress after accounting for demographic variables and living arrangements. In the final step, the unique contribution of coping strategies in predicting psychological distress was examined. The dependent variables were depression, anxiety, PTSS, and insomnia. In each regression, the predictor variables gender, age (> or < than 40 years old) and whether they were living alone (yes/no), living with children (yes/no), living with elderly individuals (yes/no), and isolated from family at any point during COVID-19 (yes/no), and time of survey were analyzed in the first step. For the second step analysis, we added the predictor variables job stress, stigma, obsession with COVID-19, fear of COVID-19, and perceived adequacy of training, protection, and support. In the final step, we analyzed coping styles (approach and avoidant coping).

## RESULTS

### Characteristics of Participants

A total of 517 EPs representing 11 institutions across 11 different states were invited to complete the survey. Participating sites included the following: the University of Mississippi Medical Center (Jackson, MS); University of Utah (Salt Lake City, UT); Keck School of Medicine of University of Southern California (Los Angeles, CA); Inspira Health Network (Vineland, New Jersey); Tulane Medical Center (New Orleans, LA); University of Alabama at Birmingham (Birmingham, AL); Henry Ford Health System (Detroit, MI); University of Texas Health Science Center (Houston, TX); University of Arizona Health Sciences (Tucson, AZ); Hennepin Healthcare (Minneapolis, MN); and Massachusetts General Hospital (Boston, MA). Three of the 11 sites were experiencing a “surge” at the time of the survey. The overall response rate using the AAPOR response rate 2 definition was 50%. This included 251 complete surveys and eight partially completed surveys (30–90% complete). Surveys were completed between October–December 2020. Respondents were 63% male and 37% female. About half of the participants were aged 30–40. Ten participants (4%) had been infected with COVID-19-. The majority (95.5%) of participants reported having adequate PPE over the prior month. Additional characteristics of the study population are shown in Table 1.

### Non-response Bias Analysis

To assess for non-response bias we compared early respondents to initial non-respondents across all mental health outcomes, based on the assumption that late respondents were similar to non-respondents.^31^ When comparing early respondents and initial non-respondents, we found no significant differences in levels of depression, anxiety, PTSS, or insomnia. The characteristics of early respondents and initial non-respondents are presented in Appendix 3. Furthermore, the proportion of female respondents in this sample (37%) is consistent with the proportion of academic EPs nationwide who are female (37%).^34^

### Construct Validity and Internal Consistency of Measures

Supporting the validity of these measures, fear of COVID-19, obsession with COVID-19, perceived stigma, and job stress were linked to anxiety, depression, and insomnia in the expected directions. As predicted, obsession with COVID-19 and fear of COVID-19 showed slightly stronger associations with anxiety than with depression. A comprehensive correlation matrix can be found in Appendix 4. Internal consistency was acceptable across measures: job stress (α = .65); perceived stigma (α =.79); obsession with COVID-19 (α =.80); fear of COVID-19 (α = .87); and training, protection, and support (α =.87).

### Mental and Behavioral Health Outcomes

Based on a single-item measure of the overall impact of the pandemic, 85% of participants reported that COVID-19 has had some negative impact on their mental health. The level of impact COVID-19 has had on EP mental health was described as large (12%); moderate (31%); and small (40%). Compared to how they felt pre-COVID-19, participants reported feeling more stressed (31%), lonelier (26%), more anxious (25%), more irritable (24%), and sadder (17.5%) (Table 2). The majority (71%) reported that their fear and anxiety about COVID-19 has at least “somewhat decreased” compared to when the outbreak started. Results from the OCS scale show that obsessive/maladaptive thinking related to COVID-19 was found in 12.5% of the sample. Responses to the Fear of COVID Scale indicate that fear of COVID-19 was common. Participants reported experiencing fear of being infected with COVID-19 (70%), fear of infecting others (77%) and fear of family being infected (84%). Specific item responses to the OCS scale and Fear of COVID scale can be found in Appendix 5.

Compared to the six months before COVID-19, participants who were exercising at least three days a week decreased slightly from 69% to 56%. The number of participants reporting daily alcohol use nearly doubled over the same period from 8% to 15%. Participants reporting some level of burnout increased from 16% to 41% (Table 2). Based on a single item, 14% of participants reported that their experiences working during COVID-19 had made them wish they had chosen a different specialty. Based on established cutoff scores and diagnostic algorithms, the prevalence of mental health conditions among the sample was 17% for depression, 13% for anxiety, 7.5% for PTSD, and 18% for insomnia.

Measures of organizational variables showed that increases in work-related stress (66%) and workload (63%) were prevalent. While most participants felt they had adequate training to work in the ED during COVID-19, only half felt appreciated and supported by their employer. Feeling stigmatized because of their work was also common (56%) (Table 3).

Table 4 displays the association between pandemic-related factors and psychological distress. Mann-Whitney U tests showed that EPs who reported isolating from family had significantly higher levels of depression (*P* < .001, effect size = .21); anxiety, (*P* = .003, effect size = .19); PTSS (*P* = .004, effect size = .18); and insomnia (*P* = .002, effect size = .19). Anxiety levels were higher among EPs who reported lacking access to PPE (*P* = .006, effect size = .17) and staffing shortages (*P* = .003, effect size = .19) (Table 3). Experiences of PTSS were higher among EPs who reported ventilator shortages (*P* = .001 effect size = .21). Gender, age, and geographical region were not associated with levels of anxiety, depression, PTSS, or insomnia.

Positive effects of COVID-19 were also reported (Table 5). Overall, 84% were at least slightly satisfied (vs dissatisfied) with their current job. The majority of participants included feeling at least “a little” more appreciated by patients and society (65%), having a greater appreciation (74%) and enthusiasm (44%) for the job, and feeling an increased sense of togetherness among colleagues (87%).

### Predictors of Mental Health Concerns (Table 6)

Next, we examined for characteristics independently associated with four mental health concerns. Models were examined in a series to identify variation in individuals’ mental health concerns that were attributable to basic individual factors (demographics, living arrangements), individual and organizational challenges related to COVID-19 (eg, fear of COVID, job stress, PPE access), and coping styles.

### Depression

We found that 9% of variation in individuals’ likelihood of depression was explained by basic individual factors, with isolation from family and later time of survey completion significantly associated with likelihood of depression symptoms. After accounting for basic individual characteristics, an additional 18% of variance in depression symptoms was explained by challenges related to COVID-19, with isolation from family, later time of survey completion, living alone, job stress, and obsession with COVID-19 significantly associated with likelihood of depression symptoms. After accounting for both basic individual factors and challenges related to COVID-19, coping behaviors predicted an additional 19% of the variance. The complete model explained 46% of the variance in depression. In the final model, living alone, isolating from family, job stress, and avoidant coping were significant predictors.

### Anxiety

We found that 11% of variation in individuals’ likelihood of anxiety was explained by basic individual factors, with female gender, living with children, later time of survey, and isolation from family significantly associated with anxiety symptoms. After accounting for basic individual characteristics, an additional 26% of variance in anxiety symptoms was explained by challenges related to COVID-19, with isolation from family, later time of survey completion, job stress, obsession with COVID-19, and fear of COVID-19 significantly predicting anxiety symptoms. After accounting for both basic individual factors and challenges related to COVID-19, coping behaviors predicted an additional 17% of the variance. The complete model explained 54% of the variance in depression. In the final model, female gender, living with children, later time of survey completion, job stress, and avoidant coping were significant predictors.

### Post-traumatic Stress Symptoms

We found that 7% of the variance in PTSS was explained by basic individual factors, with those who isolated from family and who took the survey later in time reporting higher levels of PTSS. After accounting for basic individual characteristics, an additional 19% of variance in PTSS was explained by challenges related to COVID-19, with isolation from family, job stress, and obsession with COVID-19 significant predictors. After accounting for both basic individual factors and challenges related to COVID-19, the addition of coping behaviors predicted an additional 21% of the variance in PTSS. The overall regression model predicted 47% of the variance in PTSS. In the final model, isolation from family, job stress, and avoidant coping were significant predictors.

### Insomnia

We found that 9% of the variance in insomnia symptoms was explained by basic individual factors with isolation from family and living with an elderly individual significantly predicting insomnia scores. After accounting for basic individual characteristics, an additional 9% of variance in insomnia symptoms was explained by challenges related to COVID-19, with age over 40, isolation from family, and obsession with COVID-19 significantly predicting insomnia scores. After accounting for both basic individual factors and challenges related to COVID-19, the addition of coping behaviors predicted an additional 6%. The complete model explained 24% of the variance in insomnia. In the final model, age over 40, isolating from family, and avoidant coping were significant predictors.

### Supplemental Analysis of Coping Strategies

The most commonly used coping strategies among participants were acceptance, use of emotional support, planning, and self-distraction. We conducted four additional multiple regression analyses to examine which of the 14 specific coping strategies were associated with depression, anxiety, PTSS, and insomnia. Overall, results suggest that use of behavioral disengagement, self-blame, and venting were significant predictors of psychological distress. Humor and positive reframing were associated with lower levels of psychological distress. See Table 7.

## DISCUSSION

Despite recent attention to COVID-19’s impact on the mental health of healthcare workers, this is the first nationally representative multisite study to examine its effect on US EPs. We found high levels of psychological distress due to the COVID-19 pandemic, but we also identified some positive effects from the pandemic. We also explored coping strategies that EPs used. Overall, 85% of our participants reported some negative impact on their mental health due to the pandemic. Compared to pre-pandemic levels, EPs were, on average, drinking alcohol more frequently, exercising less, spending less time with friends and family, and feeling more stressed, lonely, and anxious. This increase in negative effects is in line with many recent studies of healthcare workers in the time of COVID-19.^3^–^10^,^12^–^14^,^35^

Regarding specific outcomes, we found that a subset of individuals reported clinically elevated levels of insomnia (18%), depression (17%), anxiety (13%), and PTSD (7.5%). At first glance these prevalence rates may appear lower than rates of mental health concerns found in existing COVID-19-related studies. Several COVID-19-related studies have applied much lower cutoff scores (eg, PHQ-9 cutoff of 5 vs 10) and brief screening tools,^14^,^35^–^37^ which can lead to overestimates of prevalence rates. Rather than focusing on a narrow range of factors, this study adds to the literature by taking a comprehensive look at the impact of numerous individual (eg, demographic, fear of/obsession with COVID-19, coping strategies) and organizational (eg, practice setting, PPE, communication from leadership) factors as they relate to psychological distress.

Throughout this pandemic, EPs have demonstrated resilience and the ability to adapt to a rapidly changing medical environment. Nonetheless, existing studies tend to focus on pathologizing EPs rather than highlighting factors that contribute to their resilience. This is not to suggest that the subset of EPs who are experiencing mental health concerns should be ignored. Rather, attention should also be focused on the vast majority of EPs who are not reporting high levels of distress despite the repeated day-to-day exposure to numerous stressors. In fact, compared to a sample of the general US adult population, EPs in the current study were reporting two times lower levels of anxiety and depression than the general population.^38^ This was further echoed in the positive outcomes questions included in our survey in which 57% of respondents felt an increased sense of togetherness and cooperation among colleagues. Additionally, the majority of respondents reported feeling more appreciated by society. A little less than half of the respondents reported having a greater appreciation for the value of his/her job, while one-third reported having greater job satisfaction as well as feeling more appreciated by patients.

In terms of individual variables, coping strategies were found to play a major role in predicting or protecting against negative impacts on mental health. Engaging in avoidance coping strategies, in particular, was found to be the strongest predictor of psychological distress across all of the individual, organizational, and pandemic-related factors examined. Avoidance coping strategies include denial, substance use, venting, behavioral disengagement, self-distraction, and self-blame. When looking at the coping strategies individually, behavioral disengagement emerged as a significant predictor of all four negative mental health outcomes. Venting and engaging in self-blame were also significant predictors of elevated depression, anxiety, and PTSS in our population. Of the “Approach” coping strategies, use of “planning” as a coping response was significantly related to both depression and anxiety. Considering the uncertainty of COVID-19, it is understandable that a typically adaptive coping strategy (planning) was rendered ineffective during the outbreak. Positive reframing was also significantly negatively correlated with depression and anxiety in our population which helps explain why so many physicians reported experiencing positive outcomes from COVID-19.

Humor, which is not considered an approach or avoidance strategy, was significantly negatively correlated with three of four of the main dependent variables (depression, anxiety, PTSS). Finding ways to incorporate humor in wellness interventions, staff meetings, education sessions, and even during shifts, may be a critical strategy not receiving enough formal attention. As a whole, these findings underscore the importance of offering individual-level interventions designed to promote the use of adaptive coping strategies and identifying at-risk colleagues who may be using maladaptive coping strategies.

Organizational factors also played a significant role in predicting physician distress. In prior studies addressing healthcare worker concerns during the COVID-19 pandemic, clinicians cited lack of PPE and isolation from family as major sources of anxiety.^16^,^37^ Our findings confirmed that both lack of access to PPE and isolation from family were positively correlated with increased levels of psychological distress including depression, anxiety, PTSS, and insomnia. Higher levels of psychological distress were more common among individuals who reported experiencing PPE, ventilator, and/or staffing shortages at any point in time over the course of the pandemic.

In terms of PPE, current access to PPE was not an issue for the vast majority of the participants during the time period of this study (October-December 2020) with 95.5% of respondents reporting that they had access to adequate PPE. Nonetheless, 54.8% of physicians reported that they did not have adequate access to PPE prior to the survey, and staffing shortages were also extremely common with 73.2% of respondents reporting shortages. Both limited access to PPE (at any point during the pandemic) and staffing shortages were associated with higher levels of psychological distress. In addition, physicians who were isolated from their families experienced higher levels of anxiety, depression, PTSS, and insomnia. Our findings emphasize the need for organizational support for those separated from their families via resources such as housing and/or childcare. Increases in workload and increased job stress also had positive associations with anxiety, depression, and PTSS. Taken together, these findings highlight the importance of organizations supporting their physicians by ensuring adequate resources, staffing, and support during times of crisis.

## LIMITATIONS

Several limitations of this work deserve consideration. First, participants were a convenience sample of physicians from 11 hospitals who were identified based on known contacts at those sites; therefore, results may not be representative of the entire EP population. By limiting the number of participating programs (rather than distributing via listserves) we were able to maximize our response rate. Second, surveys were taken at a single point in time. Given the dynamic nature of the pandemic, physicians may have taken the survey before, during, or after a surge of patients. While we attempted to assess for this, these differences could have affected results. Similarly, longitudinal data were not available to assess how physicians responded to dynamic changes.

Third, the survey was targeted toward EPs at academic medical centers, and generalizability to community or rural sites is unknown. Fourth, while the hypotheses of the study were not explicit, a Hawthorne effect may have been present. Furthermore, despite the strength of the instruments used, it is possible other measures could have yielded different results. Finally, although many would consider our response rate acceptable and we found no evidence of non-response bias, there was still the potential for sampling bias.

## CONCLUSION

Emergency physicians experienced high levels of psychological distress during the COVID-19 pandemic. Individuals reporting avoidant coping strategies were most likely to experience depression, anxiety, insomnia, and PTSD. In contrast, humor and positive reframing were effective coping strategies for physicians. Strategies focusing on positive work-related experiences during the pandemic such as increased feelings of societal value or appreciation and increased sense of camaraderie with colleagues may be of value. These findings highlight the importance of hospitals supporting physicians through offering interventions designed to promote the use of adaptive coping strategies.

## Supplementary Information











## Figures and Tables

**Table 1:** Characteristics of the emergency physicians who participated in COVID-19 survey.

Participant Characteristics	% (n)^*^
Gender	
Male	63% (163)
Female	37% (96)
Age Range	
30–40	52% (134)
41–50	30% (78)
51–60	12% (32)
>60	6% (15)
Time of Survey Completion	
October 2020	44% (114)
November 2020	38% (99)
December 2020	18% (46)
Current Living Arrangements	
Alone	11% (28)
With children	65% (169)
With elderly people	5% (13)
US Region	
South	26% (65)
Northeast	15% (39)
Midwest	21% (53)
West	38% (96)
COVID-19’s Impact on Mental Health	
No negative impact	15% (39)
Small negative impact	41% (104)
Moderate negative impact	32% (81)
Large negative impact	12.5% (32)
Depression Severity; median (IQR)	8 (4–12)
Minimal	50.5% (129)
Mild	36.5% (93)
Moderate	7% (18)
Moderate to severe	5% (13)
Severe	1% (2)
Anxiety Severity; median (IQR)	4 (2–8)
Minimal	54.5% (139)
Mild	32.5% (83)
Moderate	9% (23)
Severe	4% (10)
Insomnia Severity; median (IQR)	4 (1–7)
None	49% (123)
Subthreshold insomnia	36.45% (92)
Clinical insomnia (moderate)	13.5% (34)
Clinical insomnia (severe)	1% (3)
PTSD (specific to COVID-19)	
No PTSD	92.5% (236)
PTSD criteria met	7.5% (19)
Obsession with COVID-19	
No problematic thinking related to COVID-19	87.5% (210)
Problematic thinking related to COVID-19	12.5% (30)

*US*, United States; *COVID-19*, coronavirus disease 2019; *IQR*, interquartile range.

*PTSD*, post-traumatic stress disorder.

**Table 2:** Mental and behavioral health before and during the pandemic.

Compared with how you were doing before COVID-19, how much have you been bothered by the following:	No change	A lot more than usual	A little more than usual	A little less than usual	A lot less than usual
Feeling stressed	15.2% (39)	31.1% (80)	49.8% (128)	3.1% (8)	.8% (2)
Feeling nervous or anxious	24.5% (63)	24.9% (64)	45.9% (118)	4.3% (11)	.4% (1)
Not being able to stop worrying	45% (116)	14.4% (37)	35.8% (92)	4.3% (11)	.4% (1)
Feeling sad	43.2% (111)	17.5% (45)	35.4% (91)	3.5% (9)	.4% (1)
Feeling annoyed or irritable	24.6% (63)	24.2% (62)	48% (123)	3.1% (8)	0
Experiencing lack of motivation	37% (95)	18.7% (48)	39.3% (101)	3.5% (9)	1.6% (4)
Feeling lonely	33.9% (87)	26.1% (67)	35% (90)	3.9% (10)	1.2% (3)
How often did you do the following in the 6 months before COVID-19…	Daily	3–4 days a week	1–2 days a week	1–3 days a month	Never
Exercise	28.1% (72)	41% (105)	20.3% (52)	8.2% (21)	2.3% (6)
Get together with friends	0.8% (2)	11.8% (30)	44.5% (113)	39.4% (100)	3.5% (9)
Get together with family	11.3% (29)	4.3% (11)	28.1% (72)	46.5% (119)	9.8% (25)
Drink alcohol	7.8% (20)	16.8% (39)	34% (86)	28% (70)	15% (37)
How often did you do the following in the past month…	Daily	3–4 days a week	1–2 days a week	1–3 days a month	Never
Exercise	24.5% (62)	31.6% (80)	26.9% (68)	11.5% (29)	5.5% (14)
Get together with friends in person	ND	1.6% (4)	18.1% (46)	54.3% (138)	26% (66)
Get together with friends virtually	4% (1)	1.6% (4)	15.3% (39)	45.5% (116)	37.3% (95)
Get together with family in person	9.8% (25)	4.7% (12)	8.6% (22)	52.9% (135)	23.9% (61)
Get together with family virtually	3.6% (9)	7.1% (18)	20.6% (52)	40.9% (103)	27.8% (70)
Drink alcohol	14.5% (37)	19.9% (51)	23% (59)	23% (59)	19.5% (50)
Burnout	I enjoyed my work. I had no symptoms of burnout.	Occasionally I felt under stress, and I didn’t always have as much energy as I once did, but I didn’t feel burned out.	I was definitely burning out and had one or more symptoms of burnout, such as physical and emotional exhaustion.	The symptoms of burnout wouldn’t go away.	I felt completely burned out and often wondered if I could go on. I was at the point where I needed some changes or needed to seek some sort of help.
Burnout 6 months pre-COVID-19	16% (41)	67.7% (174)	14% (36)	1.9% (5)	0.4% (1)
Burnout past month	10.5% (27)	48.6% (125)	31.9% (82)	4.7% (12)	4.3% (11)

*COVID-19*, coronavirus disease 2019.

**Table 3:** Perceived stigma, organizational support, and job stress.

	Disagree	Neither	Agree
Training protection and support			
I believe I have had adequate training to deal confidently with the situations that I face in the ED.	15.6%	12.4%	72.1%
I am provided with the PPE that I need.	15.2%	8.0%	76.8%
I believe there was adequate training provided to me in terms of infection control procedures.	25.1%	13.1%	61.8%
I believe that changes in protocols and procedures are communicated clearly to me.	27.1%	10.0%	63%
My work provides emotional support to those who need help.	12%	23.5%	64.5%
I feel appreciated by my employer.	37.4%	14.7%	47.8%
My hospital is supportive.	29.6%	17.1%	53.4%
Job stress			
I have had an increase in workload.	25.5%	11.6%	62.9%
I feel more stressed at work.	20.4%	13.9%	65.8%
There is more conflict among colleagues at work.	53.4%	20.7%	25.9%
Perceived stigma			
People avoid me because of my profession.	30.4%	13.5%	56.2%
People avoid my family members because of my work.	50.2%	18.3%	31.5%

Note: For the purpose of this table, we combined responses of “strongly disagree” “disagree,” and “somewhat disagree” into one “Disagree” category. Responses of “strongly agree” “agree,” and “somewhat agree” were combined into one “Agree” category. The full scale was used to calculate total subscale scores.

*ED*, emergency department; *PPE*, personal protective equipment.

**Table 4:** Relationship between pandemic-related factors and psychological distress.

Participant Characteristics		Depression			Anxiety			PTSS			Insomnia		
		
	% (n)	Median (IQR)	*P*	Adj. alpha	Median (IQR)	*P*	Adj. alpha	Median (IQR)	*P*	Adj. alpha	Median (IQR)	*P*	Adj. alpha
Surge during time of survey													
Yes	27.7% (70)	5 (2–9)			4.5 (1–9.5)			8 (1.5–15)			10 (3.5–13.5)		
No	72.3% (183)	4 (2–8)	.19	.017	4 (1–6)	0.08	.017	5 (1–12)	.09	.017	7 (4–11)	.12	.007
Infected with COVID-19 (any time)													
Yes	4% (10)	7 (2–12)			5 (2–11)			9 (0–29)			10.5 (5–16)		
No	96% (241)	4 (2–8)	.37	.025	4 (1–6)	0.39	.025	6 (1–13)	.46	.025	8 (4–12)	.36	.013
Isolated self from family at any point													
Yes	24.5% (63)	6 (3–9.5)			6 (1–7)			10 (2.5–21.5)			7 (4–11)		
No	75.5% (194)	4 (1–7)	**<.001**	.006	4 (1–6)	**.003**	**.006**	5 (1–11)	**.004**	**.007**	10 (6–14)	.002	.006
Adequate PPE at work (throughout COVID-19)													
Yes	41.7% (108)	5 (3–9)			5 (2–7.5)			8 (2–15)			8 (4.5–12)		
No	54.8% (142)	4 (1–7)	.03	.007	3 (1–6)	**.006**	.008	4 (.5–10)	.01	.008	8 (4–12)	.63	.025
Staffing shortages due to COVID-19													
Yes	73.2% (183)	5 (2–8)			5 (2–7)			7 (2–15)			7 (4–11)		
No	26.8% (67)	3 (1–6.5)	.06	.01	2 (0–5)	**.003**	.007	3 (1–8.5)	.02	.01	8 (4.5–12)	.41	.017
Ventilator Shortage (throughout COVID-19)													
Yes	12% (30)	6 (4–9)			5 (4–8)			10 (5–18)			9 (6–12)		
No	88% (219)	4 (2–8)	.05	.008	4 (1–6)	.013	.01	5 (1–12)	**.001**	.006	8 (4–12)	.28	.01
Access to COVID-19 testing (throughout)													
Yes	32.4% (81)	4 (1–7)			3 (1–6)			3 (1–11)			8 (3–11)		
No	67.6% (169)	4 (2–8)	.16	.0125	4 (2–7)	.02	.0125	6 (2–14)	.04	.0125	8 (4–12)	.13	.008
Replacement of N95 masks													
At least after 1 day	57.6% (144)	5 (1.5–8)			4 (1–7)			5 (1–14)			8 (4–12)		
> 1 day or never	42.4% (106)	4 (2–8)	.62	.05	4 (1–6)	.79	.05	6.5 (1–12)	.65	.05	7.5 (4–12)	.87	.05

Note: *P* values derived from Mann-Whitney U tests. Bolded *P* values denote statistical significance.

*PTSS*, post-traumatic stress symptoms; *IQR*, interquartile range; *Adj*, adjusted; *COVID-19*, coronavirus disease 2019; *PPE*, personal protective equipment.

**Table 5:** Positive outcomes as a result of COVID-19.

Rate how much you feel you have experienced change in the area described as a result of COVID-19	A great deal	A lot	Moderate amount	A little	None at all
Feel more appreciated by my patients	4.3% (11)	7.4% (19)	22.9% (59)	38.4% (99)	27.1% (70)
Feel more appreciated by society	5% (13)	20.9% (54)	25.2% (65)	38.8% (100)	10.1% (26)
Have a greater sense of job satisfaction	1.6% (4)	8.9% (23)	20.5% (53)	33.7% (87)	35.3% (91)
Have become more enthusiastic about my job	1.6% (4)	5.4% (14)	12% (31)	24.8% (64)	56.2% (145)
Have a greater appreciation for the value of my job	6.2% (16)	16.7% (43)	22.5% (58)	28.7% (74)	26% (67)
Feel an increased sense of togetherness and cooperation among my colleagues	8.9% (23)	19% (49)	28.7% (74)	30.6% (79)	12.8% (33)

*COVID-19*, coronavirus disease 2019.

**Table 6:** Three-step hierarchical multiple regression analyses for mental health outcomes.

Outcome	PTSS			Anxiety			Depression			Insomnia		
Step	1	2	3	1	2	3	1	2	3	1	2	3
Male	.07	.01	.05	.14*	.06	.10*	.07	.02	.07	.10	.05	.09
Over 40	.01	.07	.06	−.02	.06	.05	−.01	.06	.05	.12	.18**	.17**
Living alone	−.01	.03	.02	.02	.05	.04	.12	.15*	.13*	.07	.09	.07
Living with children	.05	.05	.10	.15*	.15	.19**	.11	.11	.14*	.09	.08	.10
Living with elderly	.03	.00	.03	.05	.01	.03	.06	.02	.04	.05**	.03	.03
Isolated from family	.20**	.12*	.14*	.22**	.12*	.13*	.21**	.13*	.14*	.23*	.17*	.17*
Time of survey	.14*	.09	.06	.18**	.12*	.10*	.15*	.12*	.08	.12	.10	.08
Protection and support		−.04	−.03		−.02	−.01		−.08	−.05		−.04	−.01
Job stress		.23**	.14*		.29**	.21**		.23**	.15**		.14*	.11
Stigma		−.04	−.06		−.02	−.04		−.02	−.04		.07	.06
Obsession with COVID-19		.29**	.04		.28**	.06		.24**	.00		.17*	.06
Fear of COVID-19		.08	.04		.14*	.11		.08	.06		.04	.04
Approach coping			−.05			−.07			−.13			−.14
Avoidant coping			.58**			.52**			.56**			.30**
R squared	.06	.26	.47	.11	.37	.54	.09	.27	.46	.09	.18	.24
R square change	.07*	.19**	.21**	.11**	.26**	.17**	.09**	.18**	.19**	.09*	.09**	.06**

Note: Standardized beta coefficients are reported for comparability. Male is coded as 1, other genders = 2. Over 40 is coded as 2 and less than 40 is 1. Living with and isolation variables are coded as 1 = yes or 2 = no,

* P < .05,

** P <.01.

*PTSS*, post-traumatic stress symptoms; *COVID-19*, coronavirus disease 2019.

**Table 7:** Multiple regression analyses for coping variables predicting mental health outcomes.

			Depression	Anxiety	PTSS	Insomnia
			
	Mean	SD	β	β	β	β
Avoidant coping						
Denial	2.17	0.66	−0.08	−0.02	0.03	−0.09
Substance use	2.83	1.34	.04	0.10	0.05	0.01
Venting	3.49	1.30	0.14*	0.23**	0.20**	0.03
Behavioral disengagement	2.55	1.08	0.32**	0.27**	0.27**	0.34**
Self-distraction	4.09	1.32	0.05	0.03	−0.03	0.12
Self-blame	2.91	1.29	0.39**	0.28**	0.42**	0.12
Approach coping						
Active coping	4.28	1.90	0.11	0.15**	0.06	0.09
Positive reframing	3.85	1.46	−0.14*	−0.17**	−0.04	−0.09
Planning	4.12	1.65	0.15*	0.17*	0.09	0.12
Acceptance	5.42	1.64	−0.09	−0.06	−0.06	−0.11
Use of emotional support	4.17	1.64	−0.12	−0.02	−0.002	−0.22*
Use of instrumental support	3.58	1.49	0.02	−0.01	−0.01	0.18*
Other						
Humor	3.89	1.64	−0.12*	−0.16**	−0.16**	−0.12
Religion	3.47	1.79	0.05	−0.06	0.05	−0.09

Note: Standardized beta coefficients are reported for comparability.

* P < .05.

** P < .01.

*SD*, standard deviation; *PTSS*, post-traumatic stress symptoms.
