# Supplementary material for: Sources of Distress and Coping Strategies Among Emergency Physicians During COVID-19
Source: West J Emerg Med. 2021 Oct 27;22(6):1240–52. doi: 10.5811/westjem.2021.9.53406 (PMC8597705; doi:10.5811/westjem.2021.9.53406)
Supplement: Supplementary file 2 [file wjem-22-1240-s002.docx]

Appendix 2. **Measures Description**

| Domain | Measure |
| --- | --- |
| Demographic/Living arrangements | Basic demographic data were collected (e.g., gender, age, marital status, living arrangements, geographic location.). Participants were asked about their current living arrangements (e.g., alone, with children) and whether they had isolated from family at any point during the pandemic. |
| Current Surge | Survey champions identified whether their hospital was experiencing a surge at the time participants at their site completed the survey. Surge was defined as “either a volume or acuity demand for health care services that is beyond what normal healthcare organizations and resources could supply such that additional measures and resources had to be implemented in order to accommodate the additional demand.” |
| Time of Survey | Day of survey completion was recorded for each participant in order to capturing the day of completion relative to the time period over the course of the COVID-19 pandemic. Participants who completed the survey on the first day the survey was available (October 6th) were assigned 1. Those who completed it on the last day the survey was available (December 29th) received an 86. |
| Training, Protection, and Organizational Support over the course of COVID-19 | The adequacy of their training, protection, and organizational support was measured using items adapted from a validated measure used in study during the 2003 SARS pandemic. Items are rated using a 6-point scale (1 = strongly disagree; 6 = strongly agree). Sample items include: “Infection control procedures were adequately explained” and “Changes in protocols and procedures were communicated clearly and efficiently.” Cronbach's alpha was .87  EM Physicians were asked about access to PPE (gloves, N95 masks, eye protection, gowns, etc.) and COVID testing over the course of COVID-19 (now and ~3 months ago). Sample items include: “Do you feel you have adequate PPE to effectively perform your clinical duties?”; “Are you reusing N95 masks?”; “How often are replacement N95 masks provided (after each patient contact, after 3–5 patient contacts, after 1 day, after 3–5 days, after 1 week or greater, never).” |
| Job Stress | Job Stress was measured items adapted from a validated measure used in study during the 2003 SARS pandemic. Items are rated using a 6-point scale (1 = strongly disagree; 6 = strongly agree). Sample items include: “I felt more stressed at work” and “I have an increased workload.” Cronbach's alpha was .65 |
| Perceived Stigma and Interpersonal Avoidance | Perceived stigma associated with COVID-19 was measured using items adapted from a validated measure used in study during the 2003 SARS pandemic. Items are rated using a 6-point scale (1 = strongly disagree; 6 = strongly agree). Sample item: “People avoid me because of my profession.” Cronbach's alpha was .79 |
| Fear of COVID | The Infection subscale of the validated SARS Fear Questionnaire was used to assess fear of infecting either themselves or close family members. The participants will be asked to respond to 3 items using a 4-point Likert scale (0 = definitely false; 3 = definitely true). Cronbach's alpha for this study was .87. |
| Obsession with COVID-19 | The Obsession with COVID Scale is a 4 item measure in which participants report how often they have had disturbing thoughts about COVID-19 over the past 2 weeks (0 = not at all; 4 = nearly every day). A score ≥ 7 indicates problematic symptoms. This measure has demonstrated adequate reliability and validity. Cronbach's alpha for this study was .80. |
| Coping with COVID-19 | The Brief Cope was used to assess how participants were coping with the COVID-19 pandemic. The measure consists of 28 items that assess 14 different coping strategies across the broad domains of approach coping and avoidance coping. Approach coping is characterized by the subscales of active coping, use of informational support, positive reframing, planning, emotional support, and acceptance. Avoidant Coping is characterized by the subscales of denial, substance use, venting, behavioral disengagement, self distraction and self-blame. Two other subscales humor and religion are part of the Brief Cope but are not included as part of the calculation for the approach and avoidance subscale scores. This measure has demonstrated adequate reliability and validity. Cronbach’s alphas for this study was .88 for the approach subscale and .79 for the avoidance subscale. |
| Influence of COVID-19 on mental health and daily activities | Participants were asked to report changes in stress, anxiety, sadness, irritability, loneliness, burnout, and motivation as a result of COVID-19. Changes in substance use, social support, and exercise frequency were assessed by asking participants to report their levels in each of these areas over the past month and in the 6 months pre-COVID-19. Current and pre-pandemic levels of burnout were assessed using a single item that has been validated in a sample of health care providers. “Overall, based on your definition of burnout, how would you rate your level of burnout?” The item is rate on a 5-point scale (1 = I enjoy my work. I have no symptoms of burnout; 5 = “I feel completely burned out and often wonder if I can go on. I am at the point where I may need some changes or may need to seek some sort of help.). Bivariate correlational analyses support the construct validity of the single item burnout measure by demonstrating significant associations (p<.01) with depression (r = .32) and anxiety (.31). |
| Depression | The Patient Health Questionnaire (PHQ-9) is a reliable and valid, 9 item brief measure of depressive symptoms. Respondents rate depressive symptoms in the prior 2 weeks using a 4-point rating scale (0 = not at all; 3 = nearly every day). Higher scores indicate greater symptom severity (1-4 = minimal depression, 5-9 = moderate depression, 10-14 = moderate to severe depression, 20-27 = severe depression). A score of ≥10 has a sensitivity and specificity of 88% for major depression. Cronbach’s alphas for this study was .88. |
| Anxiety | The Generalized Anxiety Disorder-7 (GAD-7) is a reliable and valid, 7 item brief measure of anxiety symptoms. Respondents rate anxiety symptoms in the prior 2 weeks using a 4-point rating scale (0 = not at all; 3 = nearly every day). Higher scores indicate greater symptom severity (1-4 = minimal symptoms, 5-9 = mild symptoms, 10-14 = moderate symptoms, 15-21= severe symptoms). A score of ≥10 has a sensitivity of 89% and specificity of 82% for generalized anxiety. Cronbach’s alphas for this study was .92. |
| Post-Traumatic Stress | The PTSD Checklist for DSM-5 (PCL-5) is a well-validated 20-item self-report measure that assesses the DSM-5 symptoms of post-traumatic stress disorder (PTSD). Respondents indicate how bothered they are by symptoms related to COVID-19 over the past month using a scale (0 = not at all; 4 = extremely). A total score of ≥31-33 is indicative of probable PTSD. Cronbach’s alphas for this study was .96. |
| Insomnia | The Insomnia Severity Index was used to assess sleep quality and disturbances. Respondents rate the nature and symptoms of their sleep over the last month using a Likert-type scale. The measure yields a total score with higher scores indicative of insomnia: absence of insomnia (0–7); sub-threshold insomnia (8–14); moderate insomnia (15–21); and severe insomnia (22-28). Cronbach’s alphas for this study was .88. |
| Positive Outcomes as a result of COVID-19 | Several items were adapted from post-traumatic growth and meaning in work measures in order to measure positive work-related outcomes associated with COVID-19. Participants were asked to rate how much they experienced a change in each area as a result of COVID-19 items using a 5-point scale (1 = A great deal; 5 = none at all). Sample items include: “I feel more appreciated by my patients” and “I have a greater sense of job satisfaction.” These items were examined individually. |
