# Supplementary material for: Sources of Distress and Coping Strategies Among Emergency Physicians During COVID-19
Source: West J Emerg Med. 2021 Oct 27;22(6):1240–52. doi: 10.5811/westjem.2021.9.53406 (PMC8597705; doi:10.5811/westjem.2021.9.53406)
Supplement: Supplementary file 3 [file wjem-22-1240-s003.docx]

**Appendix 3.** Obsession with COVID and Fear of COVID Scales

| **Obsession with COVID Scale** | | | | | | | | |
| --- | --- | --- | --- | --- | --- | --- | --- | --- |
| **How often have you experienced the following over the last month** | **Nearly every day** | **More than 7 days** | | **Several Days** | | **Rare, less than a day or 2** | | **Not at all** |
| I had disturbing thoughts that I may have caught COVID-19 | 2.8% (7) | 5.2% (13) | | 19.4% (49) | | 39.3% (99) | | 33.3% (84) |
| I had disturbing thoughts that certain people I saw may have COVID-19 | 6% (15) | 5.6% (14) | | 22.2% (56) | | 25.8% (65) | | 40.5% (102) |
| I could not stop thinking about COVID-19 | 7.1% (18) | 3.6% (9) | | 10.3 (26) | | 19.4% (49) | | 59.5% (150) |
| I dreamed about COVID-19 | 0.4% (1) | 3.2% (8) | | 7.5% (19) | | 16.7% (42) | | 72.2% (182) |
| **Fear of COVID Scale** | | | | | | | | |
| **How often have you experienced the following over the last month** | **Definitely True** | | **Somewhat true** | | **Somewhat False** | | **Definitely False** | |
| I’m afraid I will be infected with COVID-19 | 24.2% (61) | | 46% (116) | | 15.9% (40) | | 13.9% (35) | |
| I’m worried that my family will be infected | 46.8% (118) | | 36.9% (93) | | 10.7% (27) | | 5.6% (14) | |
| I’m worried I will infect others | 44.4% (1112) | | 33.3% (84) | | 12.3% (31) | | 9.9% (25) | |
