# Supplementary material for: Sources of Distress and Coping Strategies Among Emergency Physicians During COVID-19
Source: West J Emerg Med. 2021 Oct 27;22(6):1240–52. doi: 10.5811/westjem.2021.9.53406 (PMC8597705; doi:10.5811/westjem.2021.9.53406)
Supplement: Supplementary file 4 [file wjem-22-1240-s004.docx]

Appendix 4

Construct Validity Correlation Matrix

|  | Fear of COVID | Insomnia | Depression | Anxiety | Post-traumatic stress | Training Protection Support | Job stress | Stigma |
| --- | --- | --- | --- | --- | --- | --- | --- | --- |
| Obsession | .52** | .27** | .38** | .46** | .41** | -.16* | .28** | .15* |
| Fear |  | .21** | .31** | .41** | .31** | -.18** | .33** | .26** |
| Insomnia |  |  | .66** | .55** | .46** | -.10 | .23** | .13* |
| Depression |  |  |  | .82** | .75** | -.17** | .35** | .10 |
| Anxiety |  |  |  |  | .75** | -.15* | .43** | .13* |
| Post-traumatic stress |  |  |  |  |  | -.16* | .33** | .06 |
| Training Protection and Support |  |  |  |  |  |  | -26** | -.15 |
